# Si/Ge intermixing during Ge Stranski–Krastanov growth

**DOI:** 10.3762/bjnano.5.246

**Published:** 2014-12-09

**Authors:** Alain Portavoce, Khalid Hoummada, Antoine Ronda, Dominique Mangelinck, Isabelle Berbezier

**Affiliations:** 1 CNRS, IM2NP, Faculté des Sciences de Saint-Jérôme case 142, 13397 Marseille, France; 2 Aix-Marseille University, IM2NP, Faculté des Sciences de Saint-Jérôme case 142, 13397 Marseille, France

**Keywords:** atom probe tomography, germanium islands, Stranski–Krastanov growth

## Abstract

The Stranski–Krastanov growth of Ge islands on Si(001) has been widely studied. The morphology changes of Ge islands during growth, from nucleation to hut/island formation and growth, followed by hut-to-dome island transformation and dislocation nucleation of domes, have been well described, even at the atomic scale, using techniques such as scanning tunneling microscopy and transmission electron microscopy. Although it is known that these islands do not consist of pure Ge (due to Si/Ge intermixing), the composition of the Ge islands is not precisely known. In the present work, atom probe tomography was used to study the composition of buried dome islands at the atomic scale, in the three-dimensional space. The core of the island was shown to contain about 55 atom % Ge, while the Ge composition surrounding this core decreases rapidly in all directions in the islands to reach a Ge concentration of about 15 atom %. The Ge distribution in the islands follows a cylindrical symmetry and Ge segregation is observed only in the {113} facets of the islands. The Ge composition of the wetting layer is not homogeneous, varying from 5 to 30 atom %.

## Introduction

The nucleation and growth of Ge islands on a Si(001) substrate have been the subject of numerous investigations with the aim of understanding the fundamental processes involved in the Stranski–Krastanov growth process and to produce original devices based on a Ge dot assembly [1–7]. The focus of these investigations was devoted to understanding the shape of the islands and density variations versus stress (or strain) or substrate surface modifications (e.g., patterning, Si(Ge) buffer or surfactant variations) [6–11], using characterization techniques such as atomic force microscopy (AFM), scanning tunneling microscopy (STM), transmission electron microscopy (TEM) and X-ray diffraction (XRD), as well as photoluminescence spectroscopy (PL). Consequently, the control of the Ge island shape and density, as well as the control of Ge island assembly, has significantly progressed over the last years [6,12]. However, few studies have been devoted to the understanding of the Ge island composition [13–33]. This is related to the difficulty of experimentally analyzing the composition of three-dimensional (3D) nano-objects. In general, the investigations performed on Ge dot compositions involve indirect methods, often coupled with calculations. These studies shown that Ge dots do not consist of pure Ge, but rather contain a significant amount of Si in addition. However, contradicting interpretations were made concerning the atomic distribution in the islands, where some measurements led to the conclusion that the islands are made of a Si-rich core and a Ge-rich shell, and others led to the opposite conclusion [23,26,28,30,32]. It was only very recently that the calculations made by Georgiou et al. [34] resolved this controversy, showing that formation of islands with a Si-rich core is related to near-equilibrium processes and inter-island diffusion, while formation of islands exhibiting a Ge-rich core is strain driven and kinetically limited. It is important to stress that the Ge dot composition can have a significant impact on Ge-dot-based device properties, such as electron confinement and optical properties, for example. Consequently, the measurement of the Ge island composition versus growth conditions is of great interest for: (1) understanding the fundamental processes occurring at the atomic scale during growth, and (2) the control of Ge dot composition versus growth conditions or surface state for device fabrication. In addition, despite the fact that Ge islands are known to nucleate after the deposition of 3 to 6 Ge monolayers (MLs) [1–6,35], the composition and the thickness of the wetting layer (WL) are still under discussion due to Si/Ge intermixing during growth [10–11,25].

In the present work, pulsed laser atom probe tomography (APT) has been used to quantitatively study (at the atomic scale and in the 3D space) the composition of large Ge dome islands grown by gas-source molecular beam epitaxy (GS-MBE) and buried under a Si cap [36]. APT measurements show that these islands are made of a more Ge-rich core (≈55 atom % Ge) and an increasingly Ge-deficient shell (≈15 atom % Ge). Despite the strong Si/Ge intermixing during Ge island formation, the Si cap or Si substrate/island interface is abrupt, exhibiting weak Si/Ge intermixing during Si deposition. The islands keep their usual {111} and {113} surface facets under the Si cap, and Ge segregation is observed only in {113} facets. The thickness and the Ge composition of the WL are not homogeneous and fluctuate between 1 to 4.5 nm, and between 5 to 30 atom % Ge, respectively.

## Results and Discussion

**Figure 1:**
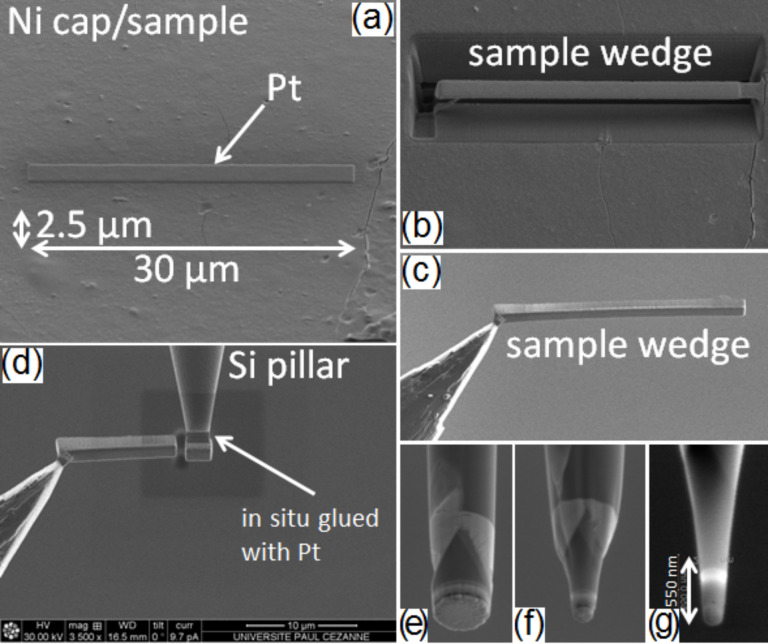
SEM images acquired during APT sample preparation in a dual-beam FIB process. The image sequence (a to f) corresponds to the chronological steps required for the fabrication of an APT tip.

The goal of this study is to quantitatively measure the composition of Ge islands in the three-dimensional space at the atomic scale using pulsed laser APT [37]. APT uses structures shaped by dual beam focus ion beam (FIB) as tips exhibiting a tip diameter between 50 nm (top of the tip) and 200 nm [38–39]. Figure 1 presents the different steps leading to the formation of APT samples by FIB. After the deposition of a Ni cap for the protection of the sample surface, the sample is loaded into a dual beam FIB. Here, an additional protective Pt layer is deposited by FIB (Figure 1a) and a wedge is cut (Figure 1b) and lifted off using an in situ tungsten finger (Figure 1c). Next, several pieces (approximately 3 × 3 µm^2^) of the sample wedge are glued onto preshaped Si pillars (Figure 1d) using FIB Pt deposition before being shaped as tips by FIB (Figure 1e–g).

**Figure 2:**
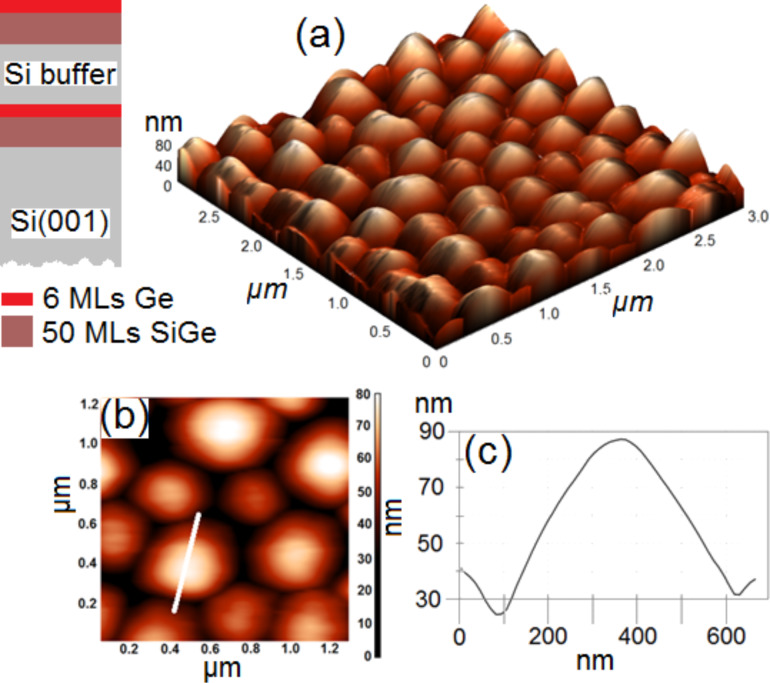
Sketch of the sample structure and AFM measurements performed on the sample surface after MBE growth: (a) 3 × 3 µm^2^ 3D image, b) 2D image, and c) height variations measured along the white line in (b).

Generally, the surface density of Ge islands is between 1 × 10^9^ to 5 × 10^10^ cm^–2^, their lateral size is between 100 and 1000 nm, and their height is between 10 and 100 nm [40]. Therefore, the difficulty lies in locating a single island in the APT sample. This is especially true for the case of small islands with a low surface density. Such islands cannot be observed by scanning electron microscopy (SEM) or FIB and the probability of shaping a tip exactly on an island is quite low. For these reasons, the island growth procedure was designed to produce large Ge islands (domes) occupying a large proportion of the sample surface. Two identical layers of islands were grown on the sample, where the first was buried by a Si layer before growing the second layer on top. Figure 2 presents AFM measurements performed on the second layer of islands, located on the surface. The island surface density is ≈6 × 10^8^ cm^–2^, and the average island height and average width are ≈72 nm and ≈430 nm, respectively.

**Figure 3:**
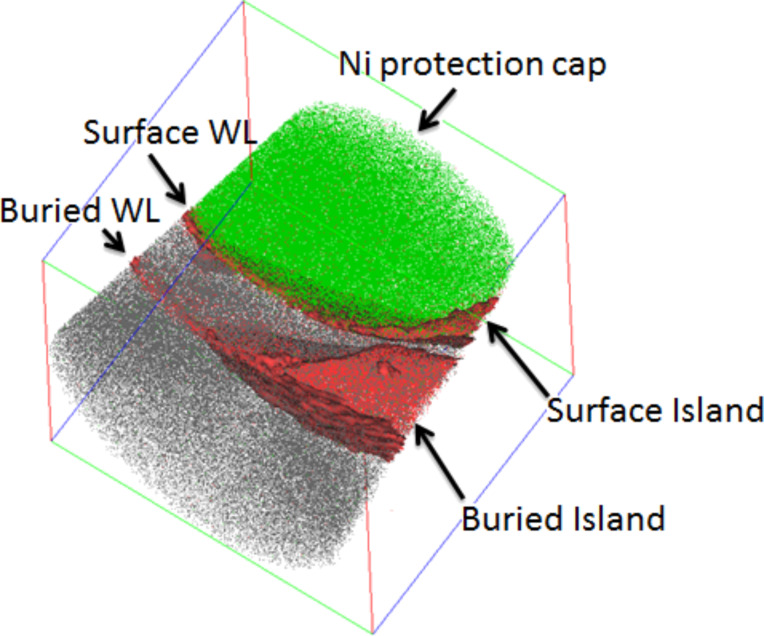
APT volume (100 × 100 × 90 nm^3^) obtained from the sample. Green, gray, red and blue dots correspond to Ni, Si, Ge and O atoms, respectively. 2 atom % Ge isoconcentration surfaces are also shown (dark red surfaces).

The sample size presented in Figure 2a corresponds to the typical size of the initial wedge piece deposited on the preshaped Si pillar (Figure 1d). The goal of steps e, f, and g presented in Figure 1e–g, is to form the apex of the tip used for APT measurements in the center of the sample in Figure 2a. Consequently, due to their lateral size, it is difficult to get an entire island in a single APT sample. However, due to the reduced distance between islands, the probability to obtain part of an island in an APT sample is high. Figure 3 presents a typical sample volume analyzed by APT. The size of the volume is 100 × 100 × 90 nm^3^. Each dot corresponds to a single atom: green, gray, red and blue dots correspond to Ni, Si, Ge and O atoms, respectively. In addition, the dark red surfaces correspond to 2 atom % Ge isoconcentration surfaces. This allows for easier delimiting of the WL and the island interfaces.

From this analysis, one can recognize the structure of the sample: the Ni cap deposited for APT sample preparation, the second layer of islands (the WL and a small part of a surface island are recognizable), the Si buffer, the first layer of islands, and the Si substrate. APT analysis allows one-dimensional (1D) atomic composition profiles to be determined in any direction in the analyzed volume.

**Figure 4:**
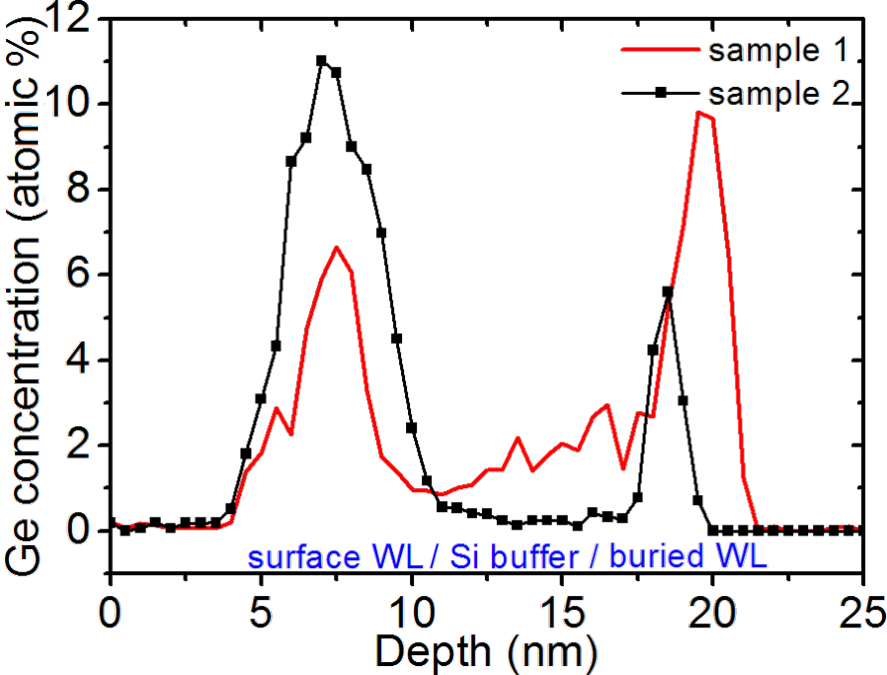
Top-down, 1D Ge concentration profiles measured between the islands in two different samples. The profiles go through the surface WL, the Si buffer, the buried WL, and end in the Si substrate.

Figure 4 shows the composition variation measured in two different APT samples through the surface WL and the buried WL in a region between islands. The surface WL and the buried WL were found to be similar (both are inhomogeneous). Their thickness and their composition vary in the sample from 1 to 4.5 nm (with an average thickness ≈2.7 nm) and from 5 to 30 atom % Ge, respectively.

**Figure 5:**
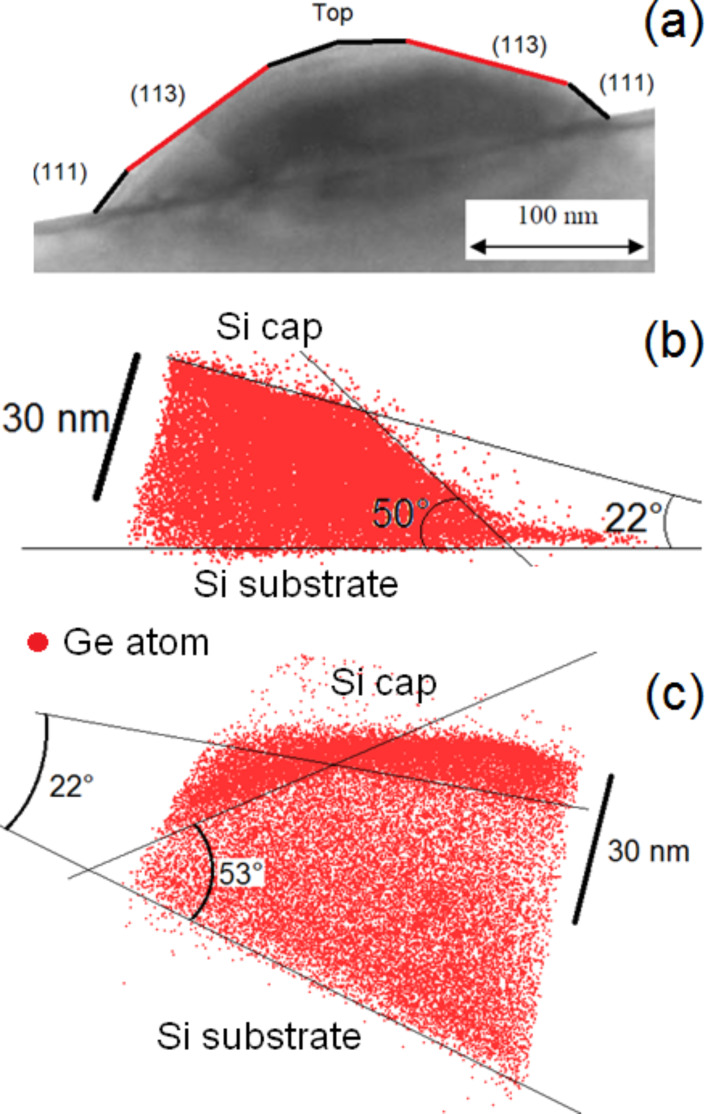
Cross-sectional TEM image of a typical dome island (a), and side-views of two different APT volumes showing only the Ge atoms they contain (b and c).

Figure 5a shows a TEM cross-sectional view of a typical dome island exhibiting {111} and {113} facets forming an angle of 54.7° and 25.2°, respectively, with the (001) surface of the Si substrate [40]. Figure 5b,c presents only the Ge atoms of buried islands in two different 3D APT volumes. As expected, the interface between the Si substrate and the islands is flat. However, one can observe facets at the island/Si cap interface. Actually, two types of facets were observed, exhibiting angles of approximately 55 ± 5° and 25 ± 5° with the Si substrate, respectively. These angles are in good agreement with the usual {111} and {113} facets of Ge dome islands [40]. The facets underneath the Si cap remained intact. In addition, as can be seen in Figure 5b,c the Si/Ge intermixing between the island base and the substrate, as well as between the island top and the Si cap is insignificant. In Figure 5c one can observe an increase of the Ge atom fraction on top of the island.

**Figure 6:**
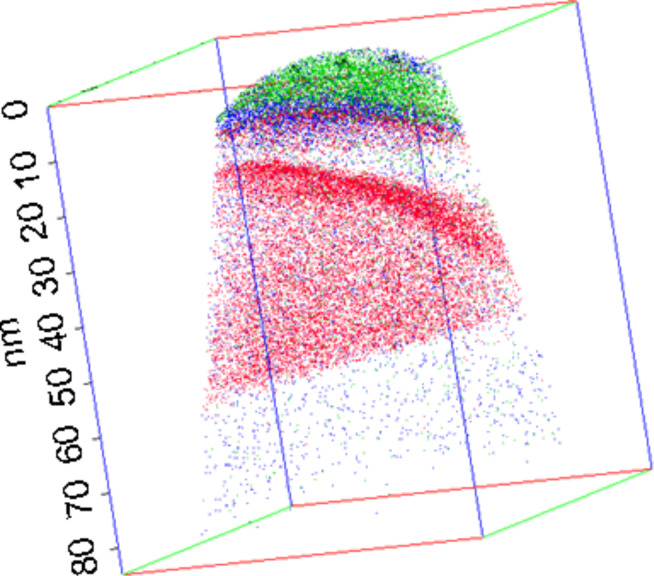
APT volume showing 2% of the Ni atoms, 5% of the Ge atoms, and 100% of the O atoms (the Si atoms are not shown). The O atoms detected in the bulk of the sample are actually due to noise.

**Figure 7:**
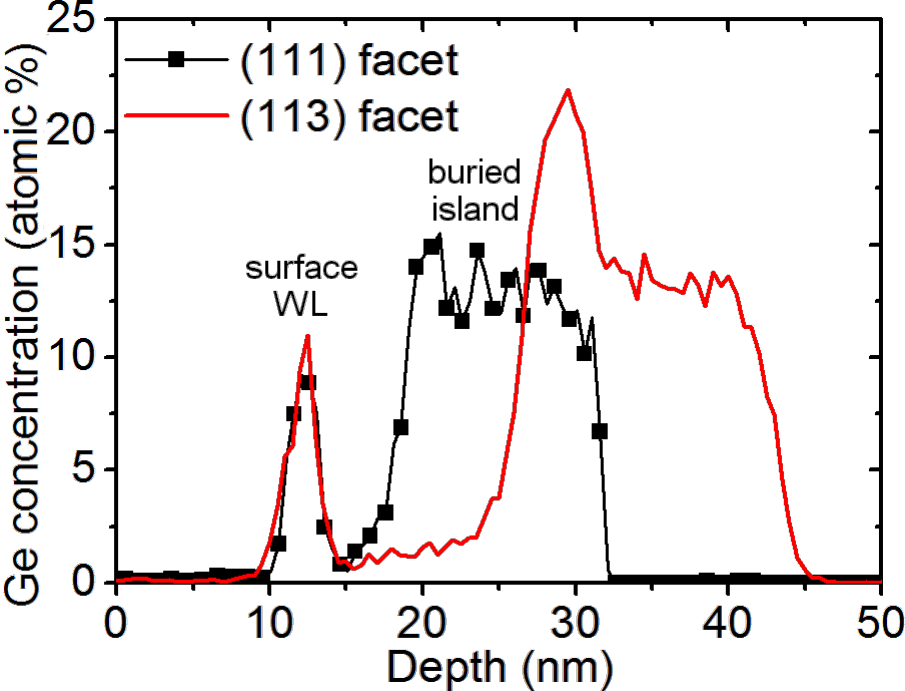
Top-down 1D Ge concentration profiles measured in two different APT volumes, one in the direction perpendicular to a (111) facet (black solid squares), and the other in the direction perpendicular to a (113) facet (red solid line).

This result is further emphasized in Figure 6 by reducing the number of atoms shown in the APT volume. Due to the superimposed effect of the 3D APT data (Figure 5 and Figure 6) it is necessary to analyze 1D composition profiles perpendicular to the facets in order to observe that Ge segregation actually only occurs on the {113} facets. For example, Figure 7 presents two different 1D composition profiles measured perpendicular to a {111} facet (squares) and perpendicular to a {113} facet (solid line). In both profiles, the surface wetting layer, with a Ge composition of about 10 atom %, can be observed. In the case of the {111} facet, the Ge concentration in the island is almost constant, at approximately 14 atom %. The {113} facet also exhibits a constant Ge concentration of approximately 14% within the island bulk. This is preceded by a region of increased Ge concentration of up to 23 atom % at the Si cap/island interface.

**Figure 8:**
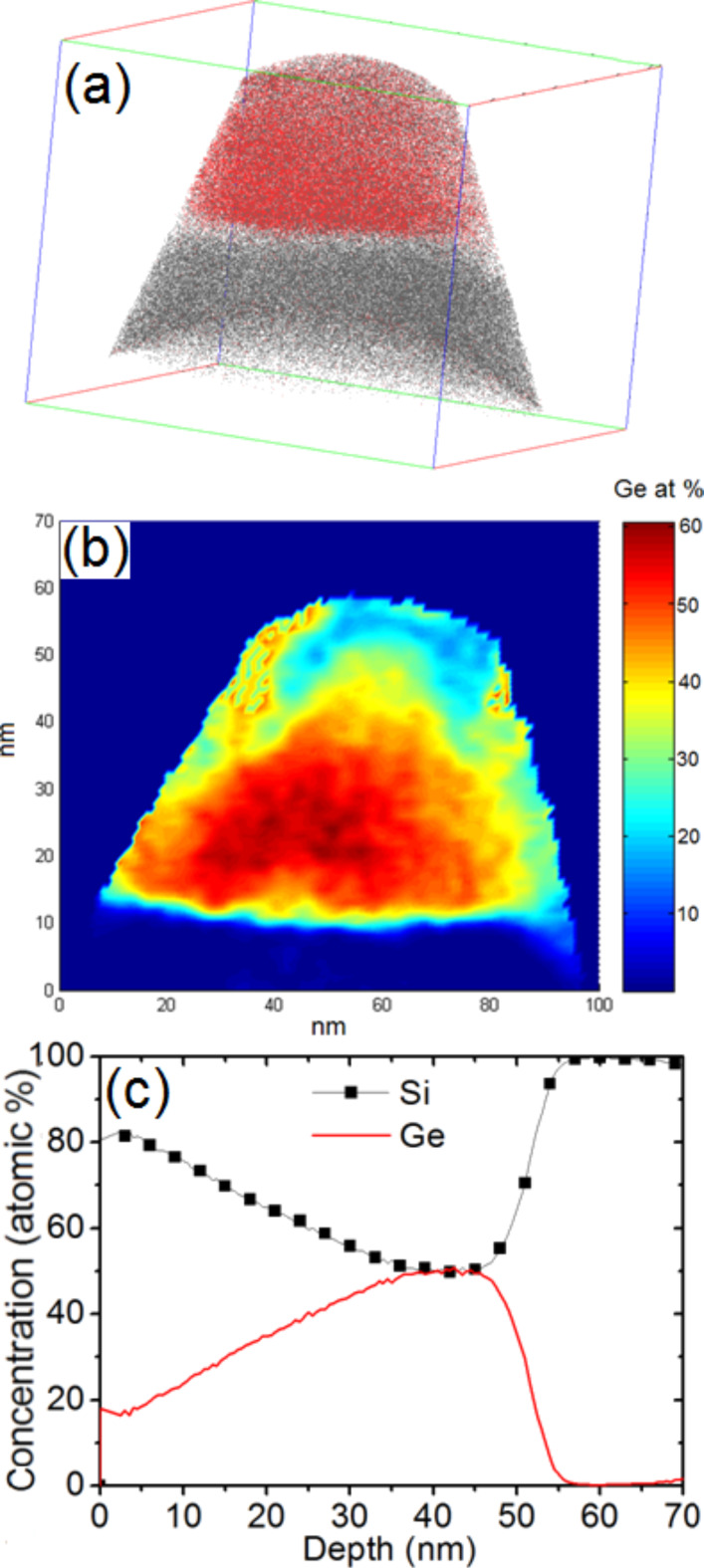
APT analysis: (a) 3D volume (120 × 120 × 100 nm^3^), (b) 2D map of the Ge concentration distribution in the center of the volume presented in Figure 8a, and (c) top-down 1D Si and Ge concentration profiles measured in the volume presented in Figure 8a.

The Ge concentration in the segregation layer of the {113} facets was found to vary from 23 to 35 atom %. Figure 8a presents another APT volume (120 × 120 × 100 nm^3^) containing the core of a Ge island. Figure 8b and Figure 8c present a 2D map and a 1D profile (top-down), respectively, of the Ge concentration in the island core. The island core is not localized in the center of the island but at the bottom, close to the Si substrate/island interface. On average, the Ge concentration in the island core is about 55 atom %.

**Figure 9:**
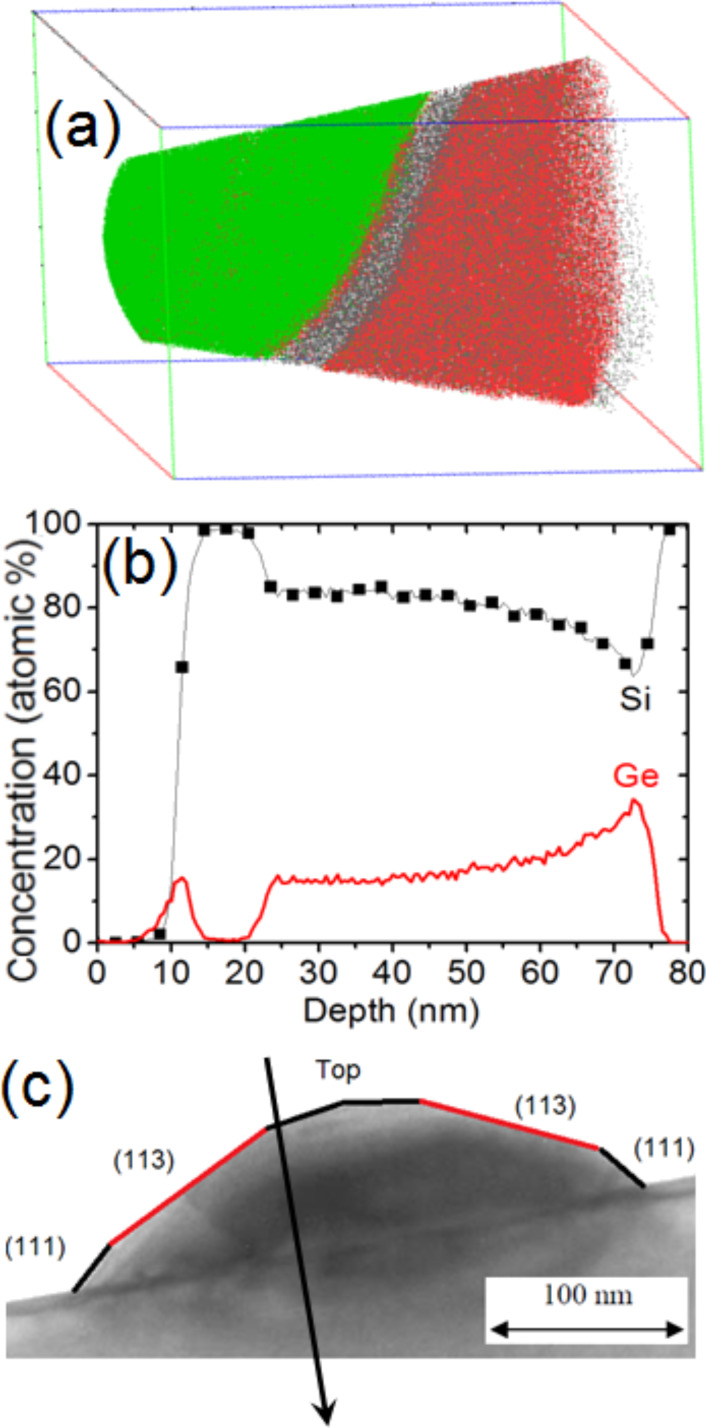
(a) APT volume (90 × 90 × 130 nm^3^) and (b) Si and Ge 1D concentration profiles measured in (a). Figure 9c indicates the direction in which the concentration profiles were measured in the island.

Figure 9a shows an APT volume (90 × 90 × 130 nm^3^) in which the 1D Ge and Si concentration profiles presented in Figure 9b have been measured. The purpose of Figure 9c is to qualitatively show where in the island the profiles in Figure 9b were measured.

**Figure 10:**
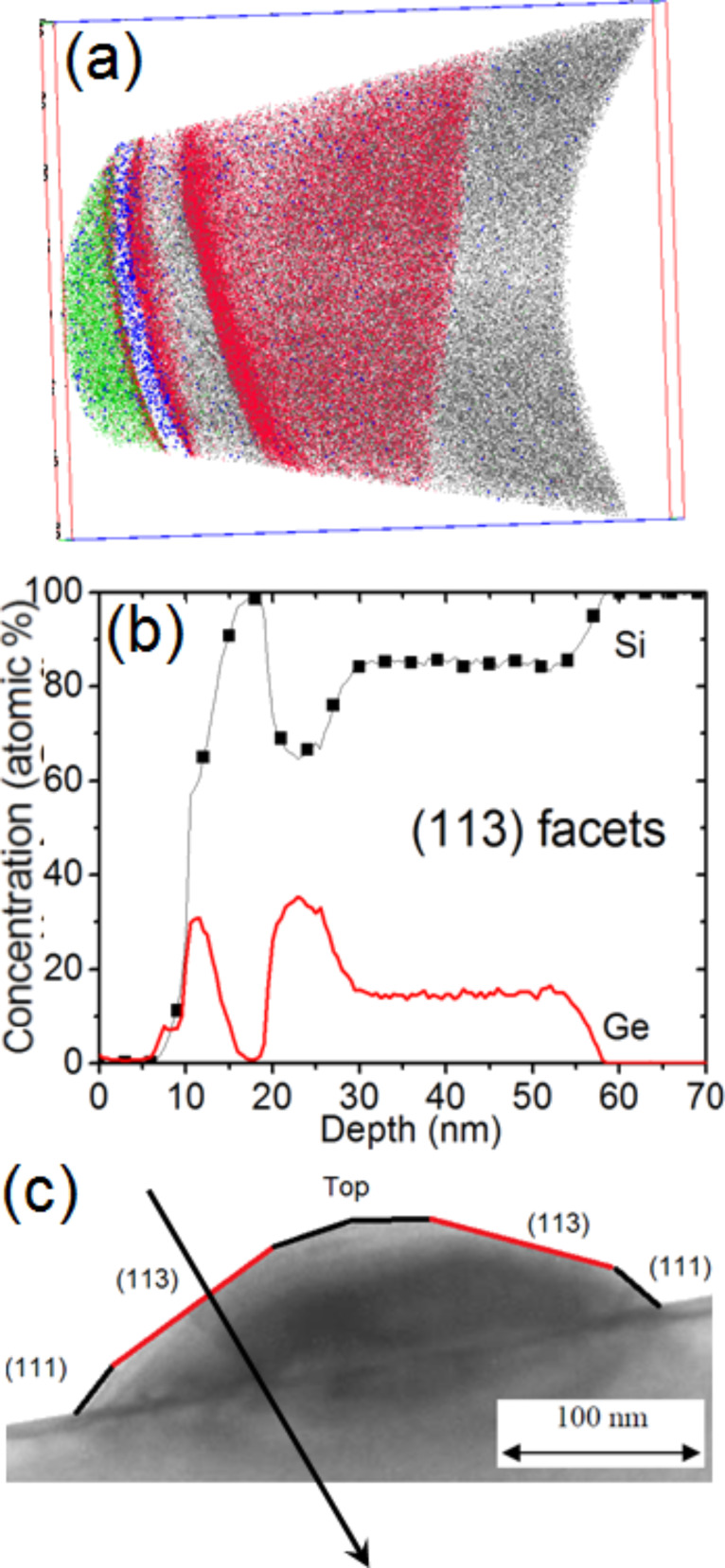
(a) APT volume (70 × 70 × 85 nm^3^) and (b) Si and Ge 1D concentration profiles measured in this volume. Figure 10c indicates the direction in which the concentration profiles were measured in the island.

We observed the surface wetting layer, followed by the Si buffer and no Ge segregation at the Si buffer/island interface. Within the island, the Ge concentration is constant (≈15 atom %) on ≈20 nm before and progressively increases until reaching the base of the island. This profile (as shown in Figure 9c) corresponds to the part of the island between the (113) facet and the top of the island. Figure 10 is similar to Figure 9, but presents an APT volume (70 × 70 × 85 nm^3^) corresponding to the part of an island just below a (113) facet. In this case, one can observe the Ge segregation at the Si cap/island interface and a constant Ge composition of ≈15 atom % in the entire island up to the island/Si substrate interface. Together, Figure 9 and Figure 10 with Figure 8 show how the Ge concentration decreases from the island core in all directions to reach a quasi-constant concentration of ≈15 atom %. It is interesting to note that the island core composition (≈55 Ge atom %) and the island shell composition (≈15 Ge atom %) appear to be independent of the size of the islands, since the APT measurements were acquired in a random distribution of islands (see Figure 2).

**Figure 11:**
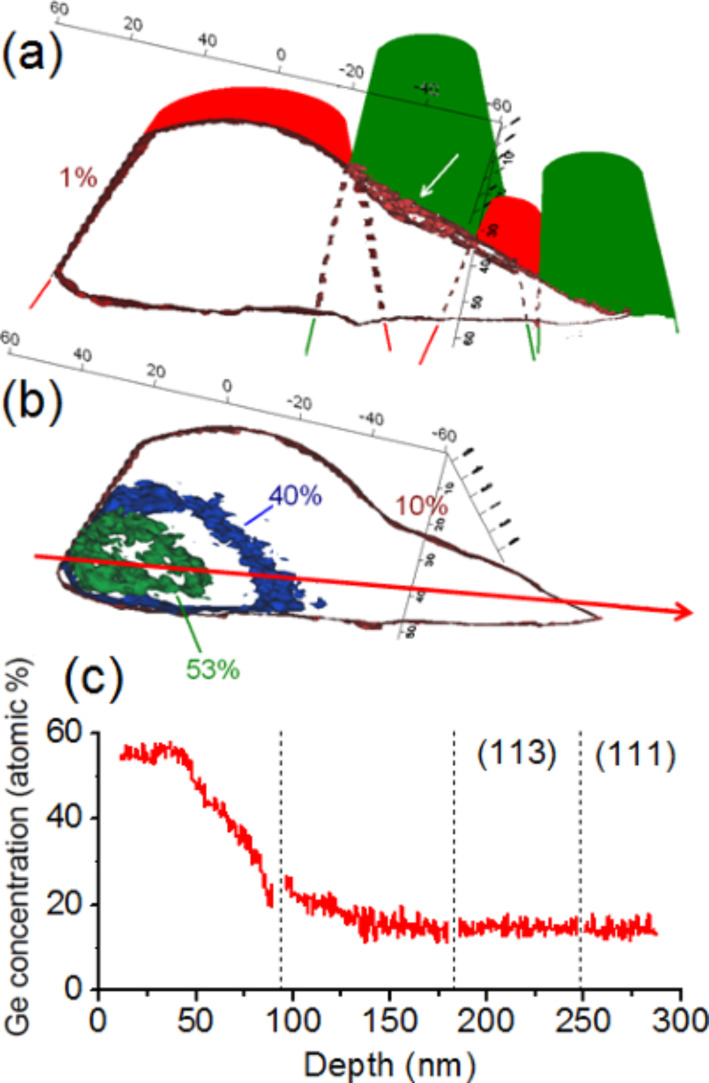
APT measurements obtained for four APT volumes (green and red surfaces) which form almost half of a Ge island: (a) Ge isoconcentration surface of 1 atom %, (b) Ge isoconcentration surfaces of 53, 40 and 10 atom %, and (c) Ge 1D concentration profile measured in the direction indicated by the red arrow in (b).

In an attempt to give an overall picture of a half-island, four APT volumes corresponding to different part of islands were combined in Figure 11. This figure shows how the different APT volumes were associated (in red and green the shape of the associated tips), as well as a Ge isoconcentration surface of 1 atom % which delimits the buried Ge island. In this image, the white arrow in the Ge segregation region is revealed by the isoconcentration surface. In order to show how the Ge concentration varies in the island, three isoconcentration surfaces are presented in Figure 11b, corresponding to the Ge concentrations of 53, 40 and 10 atom %. The Ge concentration profile along the direction shown by the red arrow in this image is presented in the Figure 11c. In this last figure, one can observe a Ge concentration plateau of ≈55 atom % in the island core and a rapid decrease of the Ge concentration reaching ≈15 atom % in the rest of the island.

The composition of Ge islands depends on the growth conditions. Ge islands exhibiting a Si-rich core were shown to correspond to growth conditions allowing for near-equilibrium states to be reached, which is more typical for the case of chemical vapor deposition [34]. Ge islands exhibiting a Ge-rich core were shown to be related to growth conditions promoting far-from-equilibrium states, controlled by kinetic processes, which is more typical for the case of MBE growth [34]. Equilibrium is reached through free energy minimization, taking into account the minimization of the surface energy, the strain energy, the alloy mixing energy and the configurational entropy [22]. In the case of a pure Ge dome island (no intermixing with Si), the stress was shown to be compressive in the interior of the island, and tensile at the edges of the island [34]. Thus, in the case of island formation close to equilibrium, the Si-rich core is attributed to the compressive conditions prevailing in the island core, and the Ge-rich outer shell is attributed to the lower surface energy of Ge and the tensile conditions prevailing at the island edges. From a kinetic point of view, it was shown that the main limiting factor of atomic redistribution is atomic diffusion (maximum diffusion length ≈1 nm normal to the facets). Atomic transport is mainly strain-driven. The diffusion path of atoms is determined by the distribution of diffusion barriers, and can explain the atomic distribution found in the islands having a Ge-rich core. In particular, Si diffusion is easier at the island border and in a thin sub-surface layer parallel to the facets (where the island composition exhibits a cylindrical symmetry), and Si atoms cannot reach the island core, while the sides of the island can experience significant strain-driven alloying. Our observations are in agreement with strain-driven and diffusion-limited atomic redistribution during MBE growth of dome islands. However, our results lie somewhat between the two extreme cases shown in [34], namely: (1) near-equilibrium islands with a Si-rich core and a Ge-rich outer-shell, exhibiting strong composition gradients in the two directions parallel and normal to the surface, and (2) kinetically-controlled islands with a core rich in Ge and border rich in Si (lateral composition variations), with negligible composition variations in the direction normal to the surface (bottom to top). Indeed, in our case, the islands exhibit a more Si-rich periphery and a more Ge-rich core, as expected for kinetically limited island growth. However, significant Ge composition gradients are found in both directions parallel and normal to the surface, with a core located at the bottom of the island, and an increase of Ge concentration can be observed on the island surface (Ge segregation on {113} facets), as expected for near-equilibrium islands.

X-ray diffraction measurements revealed the existence of atomically ordered Si/Ge domains in dome islands and the WL [41]. Ordered domains were shown to be located in a limited region of the islands, and LeGoues et al. [42] showed that Si/Ge ordering is likely linked to surface reconstruction. Recently, atom-scale Monte Carlo simulations showed that ordering domains in dome islands could indeed correspond to a surface-related phenomenon driven by surface equilibrium [43]. The calculations emphasized that ordering should be stronger in the vicinity of {15 3 23} facets and should be weak for the {105} and {113} facets. The APT instrument used for this experiment was able to measure only 40% of all the atoms in the sample, thus assessment of atomic ordering would be difficult to evidence in the APT volumes. In addition, {15 3 23} facets (contact angle ≈36°) and {105} facets (contact angle ≈11°, i.e., the facets on top of the island in Figure 5a) were not clearly identified in our measurements. However, one can note that surface segregation usually involves only one to two atomic planes, while the Ge increase observed on the {113} facets of the islands seems to concern a relatively thick subsurface layer of several nanometers in depth, as can be seen in Figures 5c, 6, 7, and 10. Thus, considering that ordered domains are due to a surface effect located in a limited region, the APT measurements suggest that Si/Ge ordering takes place at the {113} facets.

## Conclusion

Pulsed laser APT revealed the Si and Ge atomic distributions in large Ge dome islands buried by Si. The bulk composition of the islands exhibits a cylindrical symmetry. The islands are composed of a ≈55 atom % Ge core located close to the Si substrate/island interface, surrounded by a shell containing ≈15 atom % Ge. Between the islands, the thickness of the WL and its composition are not homogeneous, varying between 1 and 4.5 nm (average thickness ≈2.7 nm) and between 5 and 30 atom % Ge, respectively. The Si/Si–Ge interface is abrupt, and the islands maintain their equilibrium {111} and {113} facets under the Si cap. Ge segregation is observed only in the {113} facets, with a Ge accumulation up to 23–35 atom %. These results are in agreement with recent calculations showing that the composition of Ge islands grown by MBE should be mainly driven by strain minimization and limited by atomic diffusion kinetic barriers.

## Experimental

The sample was grown in a VG Semicon gas source MBE chamber using disilane and germane, exhibiting a base pressure in the 10^–11^ mbar range. The Si(001) substrate was chemically cleaned using a modified Radio Corporation of America (RCA) process before introduction in the MBE setup. First, the disilane gas was introduced into the growth chamber while increasing the substrate temperature (*T*) up to 850 °C in order to grow a 100 nm thick Si buffer. Then, the temperature was decreased to *T* = 700 °C and a 50 monolayer (ML) thick Si_0.7_Ge_0.3_ layer was deposited before the deposition of 6 MLs of pure Ge. These layers were then buried with a pure Si buffer before another layer (50 MLs-Si_0.7_Ge_0.3_/6 MLs-Ge) was grown (see the sketch of the sample structure in Figure 2). The entire growth was performed without interruption. Sample preparation for APT was performed using a Helios NanoLab DualBeam Ga^+^ FIB from FEI. A 100 nm thick Ni film was deposited by magnetron sputtering on each sample for protection before the samples were processed by FIB. Two types of samples were prepared: either the Ni cap was deposited without removing the surface native oxide of the sample (Figure 6, for example) or the sample was dipped in a 5% HF solution for 1 min in order to remove the native oxide (Figure 3, for example) before capping with Ni. The same results were obtained for the two types of samples. APT analysis was performed using a LEAP 3000X HR microscope in the pulsed laser mode. The analysis was carried out at 50 K, with a laser pulse frequency of 100 kHz, using a laser power between 0.5 and 0.6 nJ, corresponding to a I_Si_^2+^ /I_Si_^1+^ ratio between 100 and 10, and a I_Ge_^2+^ /I_Ge_^1+^ ratio between 7 and 2.5.

## References

[1] Voigtländer B (2001). Surf Sci Rep.

[2] Berbezier I, Ronda A, Portavoce A (2002). J Phys: Condens Matter.

[3] Teichert C (2002). Phys Rep.

[4] Stangl J, Holý V, Bauer G (2004). Rev Mod Phys.

[5] Baribeau J-M, Wu X, Rowell N L, Lockwood D J (2006). J Phys: Condens Matter.

[6] Berbezier I, Ronda A (2009). Surf Sci Rep.

[7] Portavoce A, Kammler M, Hull R, Reuter M, Ross F M (2006). Nanotechnology.

[8] Volpi F, Portavoce A, Ronda A, Shi Y, Gay J M, Berbezier I (2000). Thin Solid Films.

[9] Portavoce A, Berbezier I, Gas P, Ronda A (2004). Phys Rev B.

[10] Portavoce A, Kammler M, Hull R, Reuter M C, Copel M, Ross F M (2004). Phys Rev B.

[11] Portavoce A, Hull R, Reuter M C, Ross F M (2007). Phys Rev B.

[12] Schmidt O G (2007). Lateral alignment of epitaxial quantum dots.

[13] Regelman D V, Magidson V, Beserman R, Dettmer K (1998). Thin Solid Films.

[14] Liao X Z, Zou J, Cockayne D J H, Jiang Z M, Wang X, Leon R (2000). Appl Phys Lett.

[15] Stangl J, Daniel A, Holý V, Roch T, Bauer G, Kegel I, Metzger T H, Wiebach T, Schmidt O G, Eberl K (2001). Appl Phys Lett.

[16] Sonnet P, Kelires P C (2002). Phys Rev B.

[17] Floyd M, Zhang Y, Driver K P, Drucker J, Crozier P A, Smith D J (2003). Appl Phys Lett.

[18] Denker U, Stoffel M, Schmidt O G (2003). Phys Rev Lett.

[19] Denker U, Sigg H, Schmidt O G (2003). Mater Sci Eng, B.

[20] Sonnet P, Kelires P C (2004). Appl Phys Lett.

[21] Denker U, Sigg H, Schmidt O G (2004). Appl Surf Sci.

[22] Hadjisavvas G, Kelires P C (2005). Phys Rev B.

[23] Lang C, Cockayne D J H, Nguyen-Manh D (2005). Phys Rev B.

[24] De Seta M, Capellini G, Di Gaspare L, Evangelisti F, D’Acapito F (2006). J Appl Phys.

[25] De Seta M, Capellini G, Evangelisti F (2008). Phys Rev B.

[26] Leite M S, Malachias A, Kycia S W, Kamins T I, Williams R S, Medeiros-Ribeiro G (2008). Phys Rev Lett.

[27] De Seta M, Capellini G, Evangelisti F (2009). Superlattices Microstruct.

[28] Leite M S, Kamins T I, Medeiros-Ribeiro G (2009). Appl Phys Lett.

[R29] 29Chang, H. T.; Lee, C.-H.; Lee, S. W. The Compositional Distribution of Ge Islands Grown by Ultra-High Vacuum Chemical Vapor Deposition. In *Proceedings of the 218th Meeting of The Electrochemical Society,* Las Vegas, Nevada, Oct 10–15, 2010.

[30] Montoro L A, Leite M S, Biggemann D, Peternella F G, Batenburg K J, Medeiros-Ribeiro G, Ramirez A J (2009). J Phys Chem C.

[31] Lee S W, Lee C-H, Chang H T, Cheng S L, Liu C W (2009). Thin Solid Films.

[32] Ogawa Y, Toizumi T, Minami F, Baranov A V (2011). Phys Rev B.

[33] Biasiol G, Heun S (2011). Phys Rep.

[34] Georgiou C, Leontiou T, Kelires P C (2014). AIP Adv.

[35] Portavoce A, Berbezier I, Ronda A (2004). Phys Rev B.

[36] Portavoce A, Hoummada K, Berbezier I, Ronda A, Mangelinck D (2012). Appl Phys Lett.

[37] Miller M K, Forbes R G (2014). Atom-Probe Tomography: The Local Electrode Atom Probe.

[38] Thompson K, Lawrence D, Larson D J, Olson J D, Kelly T F, Gorman B (2007). Ultramicroscopy.

[39] Miller M K, Russell K F, Thompson G B (2005). Ultramicroscopy.

[40] Portavoce A, Ronda A, Berbezier I (2002). Mater Sci Eng, B.

[41] Malachias A, Schülli T U, Medeiros-Ribeiro G, Cançado L G, Stoffel M, Schmidt O G, Metzger T H, Magalhães-Paniago R (2005). Phys Rev B.

[42] LeGoues F K, Kesan V P, Iyer S S, Tersoff J, Tromp R (1990). Phys Rev Lett.

[43] Vantarakis G, Remediakis I N, Kelires P C (2012). Phys Rev Lett.

